# Congenital Adrenal Hyperplasia

**DOI:** 10.12688/f1000research.6543.1

**Published:** 2015-08-20

**Authors:** Phyllis W. Speiser

**Affiliations:** 1 Department of Pediatrics, Cohen Children’s Medical Center and Hofstra North Shore LIJ School of Medicine, New Hyde Park, NY 11040, USA

**Keywords:** Congenital, adrenal hyperplasia, glucocorticoids

## Abstract

Congenital adrenal hyperplasia associated with deficiency of steroid 21-hydroxylase is the most common inborn error in adrenal function and the most common cause of adrenal insufficiency in the pediatric age group. As patients now survive into adulthood, adult health-care providers must also be familiar with this condition. Over the past several years, F1000 has published numerous commentaries updating research and practical guidelines for this condition. The purposes of this review are to summarize basic information defining congenital adrenal hyperplasia and to highlight current knowledge and controversies in management.

## Introduction

In most patients with classic congenital adrenal hyperplasia (CAH), both cortisol and aldosterone production are impaired while adrenal androgen production is excessive. As a result of the lack of the vital hormones cortisol and aldosterone, patients are susceptible to potentially lethal adrenal insufficiency if untreated. Thus, emergency and critical care personnel must consider the diagnosis in patients presenting in shock. Excess androgen production, a side effect of 21-hydroxylase deficiency, causes genital ambiguity in females along with various endocrinologic, gynecologic, and reproductive complications. Men with CAH may also have reproductive and endocrine problems, most notably testicular adrenal rest tumors and oligospermia.

In this context, I will present data and arguments supporting the need for informed treatment of patients across the life span and across primary and specialty care practices. The main themes to be discussed will be prenatal and neonatal diagnosis, optimization of growth during childhood, and quality of life (QOL), including reproductive health in adult life.

## Prenatal and neonatal diagnosis

CAH is a monogenic autosomal recessive disease caused by mutations or deletions in *CYP21A2*, the gene encoding steroid 21-hydroxylase ^1, 2^. All newborns in the United States and in many developed countries are screened for 21-hydroxylase deficiency among other disorders diagnosed by obtaining heel-stick blood on filter paper ^3^. At present, the diagnosis is most often made by immunoassays for 17-hydroxyprogesterone. The assay methods have changed over time in an effort to improve the rather poor positive predictive value of these tests. Innovations in this regard have included stratifying cut-points by birth weight, introducing ratios of various analytes, and using the more specific methods of tandem mass spectrometry. In some screening programs, a second blood sample is obtained after several weeks to capture potentially missed cases. It is believed that newborn screening has significantly improved infant morbidity and mortality because of earlier diagnosis and treatment of these babies at risk for sudden death due to adrenal insufficiency ^4^. It has further been estimated that the cost-benefit ratio for screening is comparable to that of other inborn errors of metabolism for which screening has been mandated ^4, 5^.

Genotyping for screening purposes so far has not been deemed cost-effective. Rather, genotyping is most often performed when the hormonal diagnosis is in question or when genetic counseling is indicated ^6^. A common scenario is that the family with a proband affected with CAH is seeking prenatal diagnosis for a fetus. If one knows the proband’s genotype, fetal diagnosis can be accomplished during the first trimester by several approaches. Whereas in the past chorionic sampling was performed at about 10 weeks’ gestation, the novel approach of extracting fetal DNA from the maternal circulation at as early as 5 to 6 weeks holds promise for earlier anticipatory guidance ^7^. Another reproductive option for couples at risk for this and other well-characterized monogenic disorders is pre-implantation genetic screening to avoid producing a second affected child, although this procedure is considered by some to be eugenic and is quite expensive and still not widely available ^8^.

Prenatal treatment of the fetus via dexamethasone administration to the pregnant mother is potentially fraught with unknown long-term risks based on both human and animal studies ^9^. The Endocrine Society and other medical groups have deemed this practice experimental ^6^. Swedish investigators have placed a moratorium on the practice ^10^.

## Optimization of growth during childhood

The primary goal of treating classic CAH is to reduce the excess adrenal androgen production and replace the deficient hormones, namely cortisol and aldosterone. Proper treatment will prevent both adrenal crisis and ongoing virilization. Daily oral medications, including glucocorticoids, mineralocorticoids, and salt supplements, are prescribed at the time of diagnosis in infancy and titrated according to blood levels of adrenal steroids, plasma renin activity, and electrolytes measured periodically, along with annual bone age x-rays. Diets of older children and adults contain more than enough sodium, obviating the need for supplemental salt. Statural growth and weight gain are also measured regularly since overtreatment or undertreatment may be associated with inappropriate growth. The preferred treatment in children and adults is hydrocortisone, the least potent glucocorticoid ^6^. Owing to its short half-life, this drug is given in two or three daily doses. Currently being studied are newer long-acting or slowly released (or both) glucocorticoid preparations that could enhance adherence and more closely mimic physiologic cortisol secretion by the healthy adrenal cortex ^11, 12^. Retrospective systematic reviews and meta-analysis have revealed that exceeding about 17 mg/m ^2^ per day of hydrocortisone equivalents, especially during early childhood or adolescence, is associated with poorer height outcomes ^13^. During major life-threatening stress, surgery, or serious illness, patients with CAH require larger or more frequent doses (or both) of glucocorticoids and additional fluids. It is therefore crucial to educate parents of young children, and re-educate patients at the transition to adult care, about stress dosing. Unfortunately, mortality rates for CAH remain unacceptably high and are thought to be due largely to failure to appropriately deliver glucocorticoids during serious illness ^14^. Additionally, many adults with CAH are lost to follow-up, even in countries where socialized medical care is a basic benefit for all ^15^.

## Quality of life and reproductive health in adults

QOL for patients with CAH has been reported as being suboptimal to poor ^16, 17^. With CAH, as with many chronic illnesses, various factors contribute to these measures. In fact, there has been a dearth of validated CAH-specific QOL instruments. A major negative factor has been sexual function among women who underwent complex genital reconstruction by older surgical techniques ^18^. Newer approaches seem to provide better outcomes, but detailed long-term follow-up studies are lacking. Despite exposure to prenatal androgens, women with CAH most often have female core gender identity and behavior ^19, 20^. Transgender individuals have been reported but are relatively rare ^21^.

Fertility and fecundity are reduced in both men and women with CAH compared with controls ^22, 23^. Women who are more severely affected are less likely to attempt pregnancy ^24^. Additionally, adequate control of adrenal hormones, particularly follicular phase progesterone, is key in both conception and fetal retention ^24^. Pregnant women with classic CAH must be managed by high-risk practitioners and receive adjustments to steroid doses and careful fetal monitoring. Interestingly, female fetuses cannot be easily virilized by transplacental passage of maternal androgens and this is due to a very efficient placental aromatase enzyme system.

## Summary

The issues to be resolved in coming years will be reduction of fetal and neonatal morbidities and mortality associated with CAH by improved diagnostic methods as discussed above. Patient education is key to guarantee continued utilization of medical services and decreased mortality in later life. Stunted growth may be avoided by improving available steroid treatment options, thereby improving adherence. Finally, long-term outcome studies of newer genital surgery techniques will help guide management across the life span.

## Abbreviations

CAH, congenital adrenal hyperplasia; QOL, quality of life.
