# Supplementary material for: Congenital Adrenal Hyperplasia
Source: F1000Res. 2015 Aug 20;4(F1000 Faculty Rev):601. [Version 1] doi: 10.12688/f1000research.6543.1 (PMC4544382; doi:10.12688/f1000research.6543.1)
Supplement: Peer review history

# Peer review: Referee response for version 1

**DOI:** 10.5256/f1000research.7026.r10039

**Published:** 2015-08-20

**Authors:** Richard Auchus

**Affiliations:** 1 University of Michigan, Michigan, USA

I have read this submission. I believe that I have an appropriate level of expertise to confirm that it is of an acceptable scientific standard.

# Peer review: Referee response for version 1

**DOI:** 10.5256/f1000research.7026.r10038

**Published:** 2015-08-20

**Authors:** Perrin White

**Affiliations:** 1 Department of Pediatrics, University of Texas Southwestern Medical Center at Dallas, Dallas, TX, USA

I have read this submission. I believe that I have an appropriate level of expertise to confirm that it is of an acceptable scientific standard.
